# High Smad7 in the early post-operative recurrence of Crohn’s disease

**DOI:** 10.1186/s12967-020-02558-8

**Published:** 2020-10-19

**Authors:** Francesca Zorzi, Emma Calabrese, Davide Di Fusco, Elena De Cristofaro, Livia Biancone, Sara Casella, Giampiero Palmieri, Giovanni Monteleone

**Affiliations:** 1 Department of Systems Medicine, University of Rome “Tor Vergata”, Via Montpellier 1, 00133 Roma, Italy; 2 Department of Biomedicine and Prevention, Anatomic Pathology Unit, University of Rome “Tor Vergata”, Roma, Italy

**Keywords:** Inflammatory bowel disease, TGF-beta, Smad, Th1 cytokines

## Abstract

**Background:**

In Crohn’s disease (CD), one of the major inflammatory bowel disease (IBD) in human beings, there is over-expression of Smad7, an intracellular inhibitor of the suppressive cytokine TGF-β1. The aim of this study was to assess whether Smad7 over-expression occurs in the early and/or late phases of CD.

**Methods:**

Mucosal samples were taken from the neo-terminal ileum of CD patients undergoing ileocolonic resection, with or without (early CD) post-operative endoscopic recurrence, and terminal ileum of CD patients with long-standing disease undergoing intestinal resection (late CD). Smad7 was examined by immunohistochemistry and cytokine expression was analysed by flow-cytometry.

**Results:**

Before the appearance of endoscopic lesions, the mucosa of the neo-terminal ileum contained high number of Smad7-expressing cells in both the epithelial and lamina propria compartments. Transition from this stage to endoscopic recurrence was marked by persistence of high number of Smad7-positive cells, which reduced significantly in the late stages of the disease, where Smad7 expression remained, however, greater than that seen in normal controls. In samples with early lesions, Smad7 expression positively correlated with the number of interferon-γ-secreting cells.

**Conclusions:**

Smad7 induction is an early event in the inflammatory sequence occurring in CD, thus suggesting that knockdown of Smad7 can help prevent post-operative recurrence.

## Background

Crohn’s Disease (CD) is a chronic inflammatory disorder characterized by transmural and segmental lesions, which can occur in any part of the alimentary tract, even though they are more common in the terminal ileum and/or right colon. [1] Despite recent advances in the management of CD, nearly half of the patients undergo surgery within 10 years after diagnosis, mainly due to failure of medical therapy or development of local complications, such as strictures and/or fistulae. [2, 3] Ileocolonic end-to-end anastomoses and side-to-side anastomoses are the standard surgical treatments for most CD patients who undergo ileocecal resection. [4] Unfortunately, however, surgery does not cure CD, and almost all the patients will experience endoscopic recurrence at the site of anastomosis, which inevitably leads to clinical exacerbation. [5] Some demographic, lifestyle-related and clinical factors have been related to increased risk of post-operative recurrence in CD, [6, 7] but the exact basic mechanism underlying CD recurrence remains unknown. In this context, we have recently shown that the mucosa of the neo-terminal ileum after ileocecal resection is infiltrated with immune cells secreting high levels of inflammatory T-helper (Th) type-1-related cytokines before the manifestation of endoscopic recurrence (“early CD”). [8] A similar temporally regulated cytokine profile was seen in mouse models of colitis, where the early stage of the colonic inflammation was driven by Th1-cytokines. [9] These findings, together with the demonstration that blockers of tumor necrosis factor alpha (TNF-α) are useful in the management of CD recurrence [10], support the hypothesis that Th type-1-related cytokines, such as IFN-γ and TNF-α, may play a role in driving the postoperative recurrence of CD.

Although the cause of CD remains unknown, epidemiological and experimental studies support the hypothesis that CD is due to multiple environmental factors, which in genetically-predisposed individuals trigger an excessive inflammatory response directed against components of the gut microflora. [11, 12] It has also been demonstrated that defects in counter-regulatory mechanisms contribute to amplify the ongoing mucosal inflammation. For instance, in CD, there is diminished activity of transforming growth factor (TGF)-β1, a regulatory cytokine that inhibits inflammatory signals in many immune cells. [13] Such a defect is secondary to elevated levels of Smad7, an intracellular protein that binds to TGF-β receptor type I and inhibits TGF-β1-induced signalling. [14–16] Consistently, inhibition of Smad7 with a specific antisense oligonucleotide (AS) restored TGF-β1 activity and suppressed inflammatory pathways in both in vitro and in vivo models of intestinal inflammation. Phase 1 and phase 2 studies showed that knockdown of Smad7 with a pharmaceutical compound containing the Smad7 AS induced clinical and endoscopic improvement in CD patients, [17, 18] even though a recent phase 3 study was discontinued following a futility analysis showing no benefit in patients treated with such a drug as compared to those receiving placebo. [19]

The functional relevance of the TGF-β1 defects in the pathogenesis of CD is also supported by studies in mice showing that lack of the cytokine activity is sufficient to promote the development of gut inflammation. [20] Altogether, these observations raise the possibility that induction of Smad7 and consequent defective TGF-β1 activity can occur early in the sequence of molecular events that lead to tissue damage. The aim of this study was to assess whether Smad7 induction occurs early and/or late in CD.

## Methods

### Patients and samples

CD patients undergoing intestinal resection for a chronically active disease poorly responsive to medical treatment were prospectively enrolled. Four specimens were taken from inflamed, but not ulcerated, ileal mucosa of these patients. Surgical samples were collected in completed medium 30–60 min after the intestinal resection and immediately processed. After surgery, all the patients underwent ileocolonoscopy at 6 or 12 months depending on the clinical activity or presence of risk factors for severe disease (i.e. smoking habit, young age onset, rectal disease). Endoscopic recurrence was graded according to the Rutgeerts’s score (0: no lesions; 1: less than 5 aphthous lesions; 2: more than 5 aphthous lesions with normal mucosa between the lesions, or skip areas of larger lesions, or lesions confined to the ileocolonic anastomotic lining; 3: diffuse aphthous ileitis with diffusely inflamed mucosa; and 4: diffuse ileal inflammation with larger ulcers, nodules, or narrowing. Hyperaemia and oedema alone were not considered as signs of recurrence). [6] During ileocolonoscopy, 4 adjacent ileal biopsy samples were systematically taken about 10 cm above the anastomosis from the most inflamed, but not ulcerated, ileal mucosa or from macroscopically unaffected mucosa. Four biopsies were also taken in the terminal ileum, 10–25 cm above the ileo-cecal valve, of normal controls (CTR) who underwent ileocolonoscopy for irritable bowel syndrome: no endoscopic lesion was found in these patients and the ileal mucosa was histologically normal.

Each patient who took part in the study gave informed consent and the study was approved by the local Ethics Committee.

### Immunohistochemistry

All reagents were from Sigma-Aldrich (Milan, Italy) unless specified. Immunohistochemistry was performed on formalin-fixed, paraffin-embedded sections of CD patients and CTR. The sections were deparaffinized and dehydrated through xylene and ethanol and the antigen retrieval was performed in citrate buffer (pH 6.0) for 20 min in microwave. Immunohistochemical staining was performed using a rabbit anti-SMAD7 (orb11386, Biorbyt Ltd), at room temperature for 1 h*.* Immunoreactive cells were visualized using MACH4 Universal HRP-Polymer kit (Bio- care Medical, Concord, CA, USA) with 3,3′-Diaminobenzidine (DAB) (Dako North America, Carpinteria, CA, USA) as a chromogen system, according to the manufacturer’s instructions, and lightly counterstained with hematoxylin. Isotype control IgG-stained sections were prepared under identical immunohistochemical conditions as described above, replacing the primary antibody with a purified rabbit normal IgG control antibody (R&D Systems, Minneapolis, MN, USA). Both Smad7-positive epithelial cells and Smad7-positive lamina propria cells were identified by high power magnification of the immunohistochemical images and the positive cells were manually counted in 5 high power fields from each slide. Sections were analyzed by LEICA DMI4000 B microscope expressed as number of cells for high power field (hpf).

### Lamina propria mononuclear cell isolation

Lamina propria mononuclear cells (LPMC) were isolated from ileal biopsy samples and intestinal resection specimens of CD patients and CTR as described elsewhere. [21] LPMC were suspended in RPMI 1640 medium, supplemented with 10% inactivated fetal bovine serum (FBS), penicillin (P) (100 U/ml), and streptomycin (S) (100 µg/ml) (Life Technologies-GibcoCRL, Milan, Italy) at concentration of 1 million per ml and used to assess cytokine expression by flow cytometry.

### Flow-cytometry analysis

LPMC were seeded in 96-well U-bottom culture dishes and stimulated with phorbol myristate acetate (PMA) (10 ng/mL), ionomycin (1 µg/mL), and brefeldinA (10 µg/mL;eBioscience, San Diego, CA). After 5 h, cells were stained with anti-CD3-PerCP (1:50, final dilution, BD Biosciences, San Jose, CA) and fixed with 1% formaldehyde for 20′. Subsequently cells were permeabilized with 0.5% saponin in 1% BSA FACS buffer and stained with the following Abs: anti-interferon (IFN)-γ-PE (1:50, final dilution; BD Biosciences), anti–interleukin (IL)-17A–APC (1:50, final dilution, eBioscience). Appropriate isotype-matched controls from BD Biosciences were included in all of the experiments. Cells were analysed using a FACS Calibur cytometer and Cell-QuestPro software.

### Statistical analysis.

Since this was a pilot study, no calculation of the simple size was made. Statistical differences were assessed with the GraphPad Prism statistical PC program (GraphPad Software, San Diego, CA). Nonparametric data were analyzed using the Mann–Whitney U‐test for comparison between two groups or Kruskal–Wallis test for multiple comparison. Significance of correlation was determined using the Spearman non-parametric correlation.

A *p* value of less than 0.05 was considered statistically significant.

## Results

### Study population: clinical and endoscopic data

Seventeen CD patients (male, 82%) who underwent ileocolonic resection for a chronically active disease poorly responsive to medical treatment were included in this study. The demographic and clinical characteristics of the patients are shown in Table 1. Median age of the patients was 37 years (range, 21–64 years). Only one patient was smoker at the time of endoscopy. In all these patients, lesions (herein termed late/established CD) were confined to the terminal ileum. At the time of surgery, 9 patients were on steroids, and 2 of them were taking simultaneously azathioprine, while 2 patients had previously received anti TNF-α. Mucosal samples were collected from the resected ileum of 11/17 patients [8 male; median age 53 (21–69) years, median disease duration 149 (36–312) months]. In the remaining 6 patients, mucosal samples were not available as surgeries were performed in urgency.

**Table 1.** Demographic and clinical characteristics of patients

Patients characteristics	CD pts	Control pts
	n = 17	n = 5
Age years, median (range)	37 (21–64)	38 (28–55)
Sex male, N (%)	14 (82%)	2 (40%)
Smoking habits, N (%)		
Yes	1 (6%)	1 (20%)
No	16 (94%)	4 (80%)
CD duration months, median (range)	180 (12–320)	na
Age at diagnosis, N (%)		
A1: ≤ 16 years	1 (6%)	na
A2: 17–40 years	13 (76%)	
A3: over 40 years	3 (18%)	
CD behaviour, N (%)		
B1: inflammatory	0	na
B2: Stricture	11 (65%)	
B3: Penetrating	6 (35%)	
CD location, N (%)		
L1: Ileal	17 (100%)	na
Harvey Bradshaw index at time of endoscopy^a^		
Median (range)	4 (2–9)	na
Remission	13 (76%)	
Active	4 (24%)	
Rutgeerts score at 6 month after surgery^b^		
No CD recurrence (i0-i1)	4 (23%)	na
CD recurrence (i2-i4)	8 (47%)	
No endoscopy	5 (30%)	
Rutgeerts score at 12 month after surgery^b^		
No CD recurrence (i0-i1)	2 (12%)	na
CD recurrence (i2-i4)	11 (65%)	
No endoscopy	4 (23%)	
CD medication at time of endoscopy		
No medication	1 (6%)	5 (100%)
5-aminosalicylic acid	14 (82%)	0
Corticosteroids	0	0
Thiopurine alone	2 (12%)	0
TNFs alone	0	0

^a^Adapted from Harvey-Bradshaw index

^b^Adapted from Rutgeerts score

At the time of ileocolonoscopy, 14 out of 17 patients (82%) were receiving mesalamine and 2/17 (12%) patients were received thiopurines. Twelve patients underwent ileocolonoscopy 6 months after ileo-colonic resection. Of these patients, 8 (66.6%) had endoscopic recurrence (4 pts with Rutgeerts score i2 and 4 pts with Rutgeerts score i4) while in the remaining 4 (33.3%) there was no endoscopic lesion (3 pts with Rutgeerts score i0 and 1 pts with Rutgeerts score i1). Four out of these 12 (33.3%) CD patients had a clinically active disease (Harvey-Bradshow index, HBI > 5) and all of them had endoscopic recurrence (i2-i4).

The remaining 5 patients who participated in the study underwent endoscopy 12 months after the intestinal resection. One of these patients had no endoscopic recurrence i0 and the remaining 4 had endoscopic recurrence: Rutgeerts score i2 in 4 patients and i4 in 1 patient. Additionally, endoscopy was performed at 12 months in 3 out of the 4 patients who had no endoscopic recurrence at month 6 because they became symptomatic: 2 out of these 3 patients developed endoscopic recurrence (Rutgeerts score i3 in 1 patient and i2 in the other one). Endoscopy was also performed at month 12 for a worsening of symptoms in 5 patients, who had endoscopic lesions (grade i2 according to Rutgeerts) at month 6; in all these patients, the second endoscopy evidenced severe endoscopic recurrence (Rutgeerts score i3-i4) (Additional file 1: Table S1).

### Smad7+ cells infiltrate the neo-terminal ileum of CD patients independently of the presence of endoscopic recurrence

**Fig. 1:**
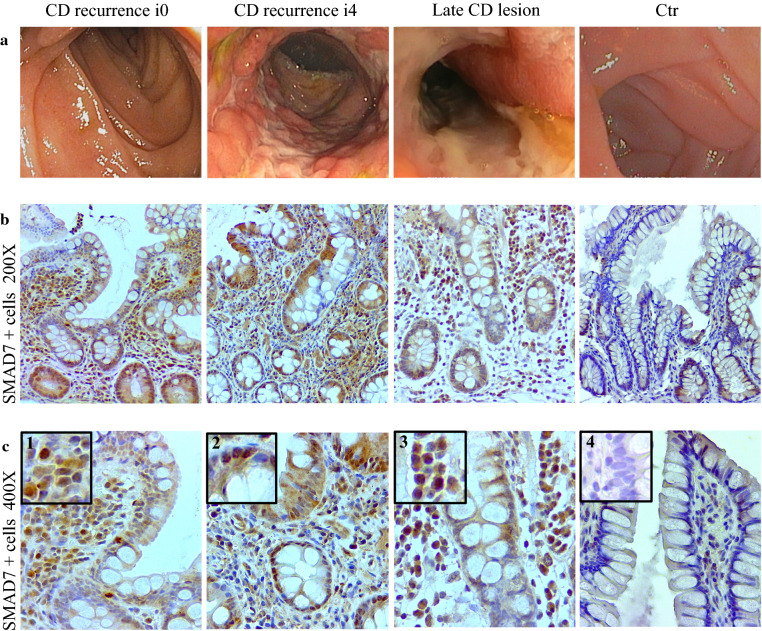
Smad7-positive cells accumulate in the terminal ileum of Crohn’s disease patients independently of the presence of endoscopic lesions**.** **a** Representative endoscopic pictures showing the distal ileum of 1 CD patient with no evidence of post-operative endoscopic recurrence (i0), 1 CD patient with severe endoscopic recurrence (i4), 1 CD patient with established (late) lesion and 1 normal control patient. **b**, **c** Representative photomicrographs showing Smad7-stained paraffin embedded sections of biopsy samples taken from ileal sections of 1 CD patient with no evidence of endoscopic recurrence (i0), 1 CD patient with severe endoscopic recurrence (i4), 1 CD patient with established (late) lesion and 1 normal control patient. Original magnifications 200X and 400X are shown in **b** and **c** respectively. Insets 1–3 in **c** show higher magnification photomicrographs; staining with control IgG is shown in the inset 4 of (**c**)

**Fig. 2:**
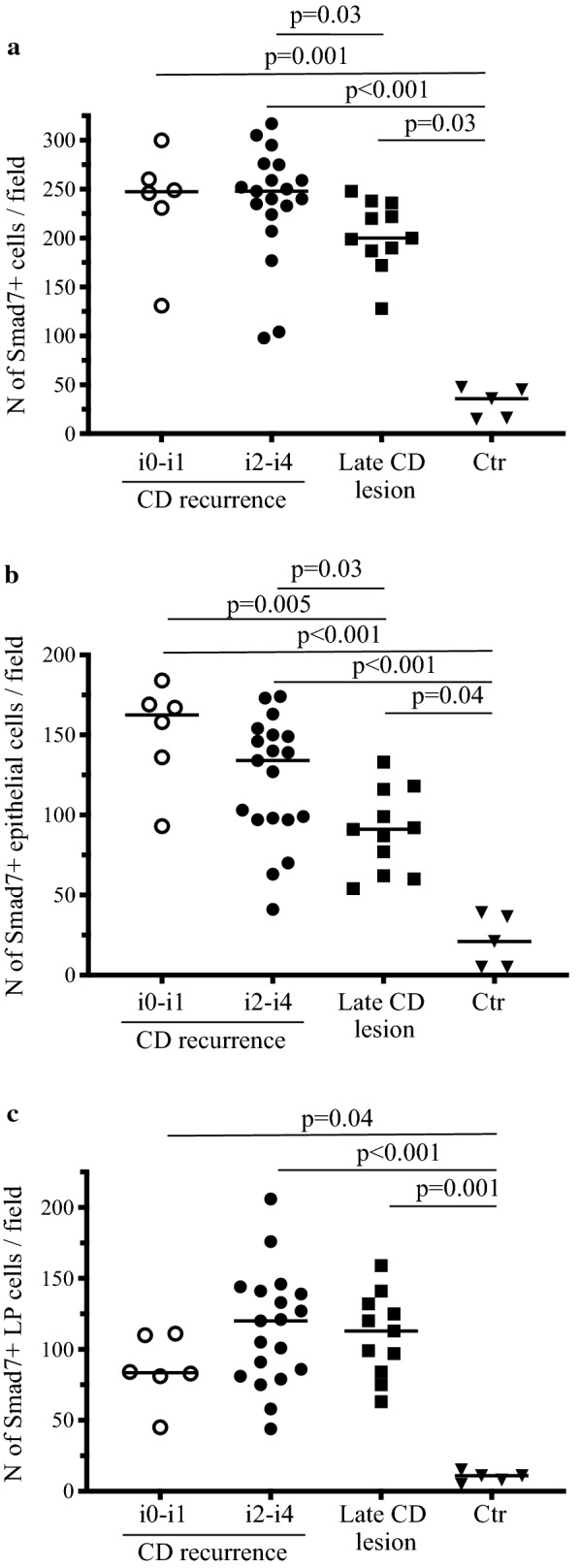
Up-regulation of Smad7 in the epithelial and lamina propria compartments of the ileal mucosa of Crohn’s disease patients. Quantification of Smad7-positive cells in the whole intestinal mucosa (**a**), epithelial (**b**) and lamina propria (**c**) compartments of 6 CD patients with no endoscopic recurrence (i0-i1), CD patients with endoscopic recurrence (i2-i4), CD patients with established lesions and normal control patients. Smad7 positive cells were manually counted in at least 5 high power fields/section of 3 independent experiments. Data indicate the individual number of Smad7 + cells in each patient; horizontal bars indicate the median values

**Fig. 3:**
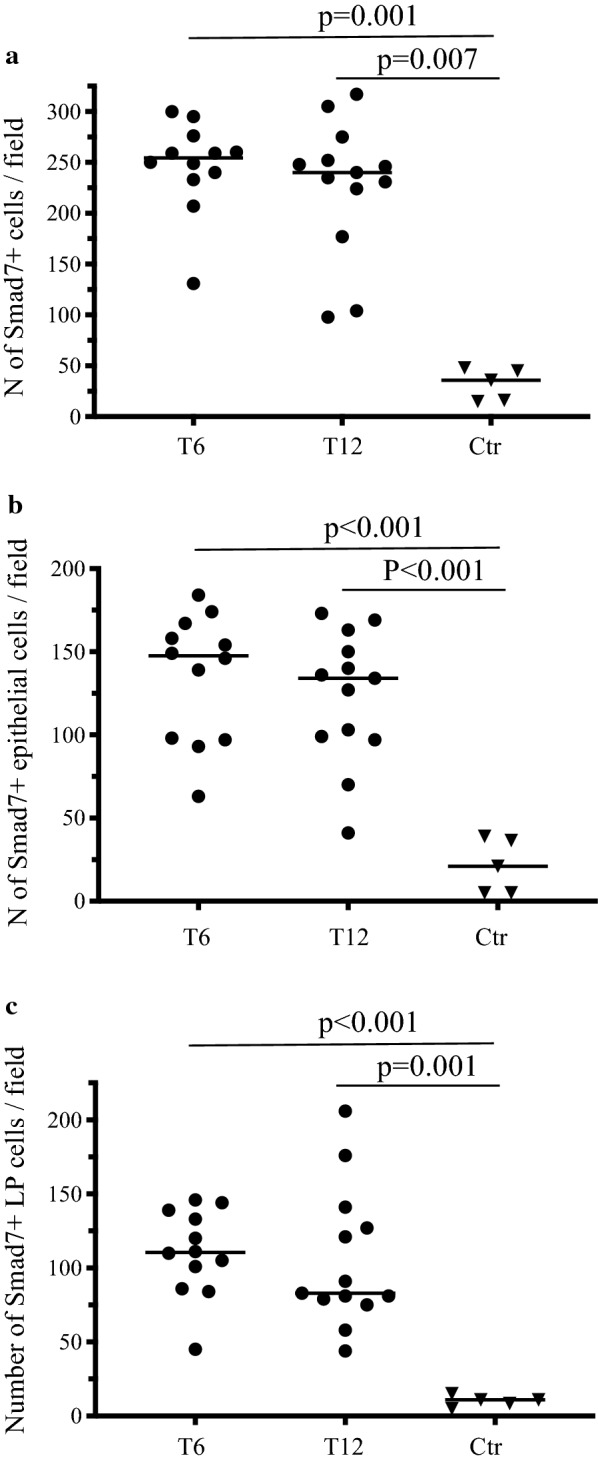
Smad7 is up-regulated in the early lesions of Crohn’s disease. Quantification of Smad7 positive cells in the whole intestinal mucosa (**a**), epithelial (**b**) and lamina propria (**c**) compartments of 12 CD patients undergoing endoscopy 6 months after ileocolonic surgery, 13 CD patients undergoing endoscopy 12 months after ileocolonic surgery and 5 normal controls. Smad7 positive cells were manually counted in at least 5 high power fields/section of 3 independent experiments. Data indicate the individual number of Smad7 + cells in each patient; horizontal bars indicate the median values

Following ileocolonic resection, CD lesions almost invariably develop in the previously uninflamed mucosa of the neo-terminal ileum proximally to the ileocolonic anastomosis. [5, 6] This post-operative state is a useful setting to investigate molecules, which could be relevant for triggering and/or amplifying the tissue-damaging immune response. Therefore, we collected biopsies from CD patients with or without endoscopic recurrence and examined the expression of Smad7 by immunohistochemistry. Smad7-positive cells were more evident in CD samples than in CTR. Such cells were abundantly present in CD mucosal samples independently of the presence of endoscopic recurrence in both the epithelial and lamina propria compartments (Fig. 1). Notably, in CD sections, Smad7 accumulated in the cytoplasm and nucleus of both epithelial cells and LPMC (Fig. 1c). Analysis of the Smad7-positive cells in each CD and CTR section revealed that the number of Smad7 + cells was significantly higher in samples taken from the neo-terminal ileum of CD patients without endoscopic recurrence, CD patients with endoscopic recurrence and patients with established disease than in normal CTR (Fig. 2a). Interestingly, the total number of Smad7 + positive cells per sample was higher in the groups of CD patients without endoscopic recurrence and patients with endoscopic recurrence than in the group of patients with established disease (Fig. 2a). This finding was also evident when analysis of the Smad7 + cells was restricted to the epithelial compartment (Fig. 2b). In the lamina propria compartment, Smad7 + cells were more abundant in CD samples than in CTR with no significant difference among the 3 groups of CD patients (Fig. 2c). Further analysis at the 2 time-points (i.e. 6 and 12 months after ileocolonic resection) selected to investigate the occurrence of the endoscopic recurrence showed no significant difference in the number of Smad7-expressing cells (Fig. 3).

Overall, these data indicate that, even in the absence of endoscopic lesions, the mucosa of the neo-terminal ileum of CD patients is marked by accumulation of Smad7-positive cells.

### In the early stage of CD inflammation, expression of Smad7 correlates with the number of interferon-γ-secreting cells

**Fig. 4:**
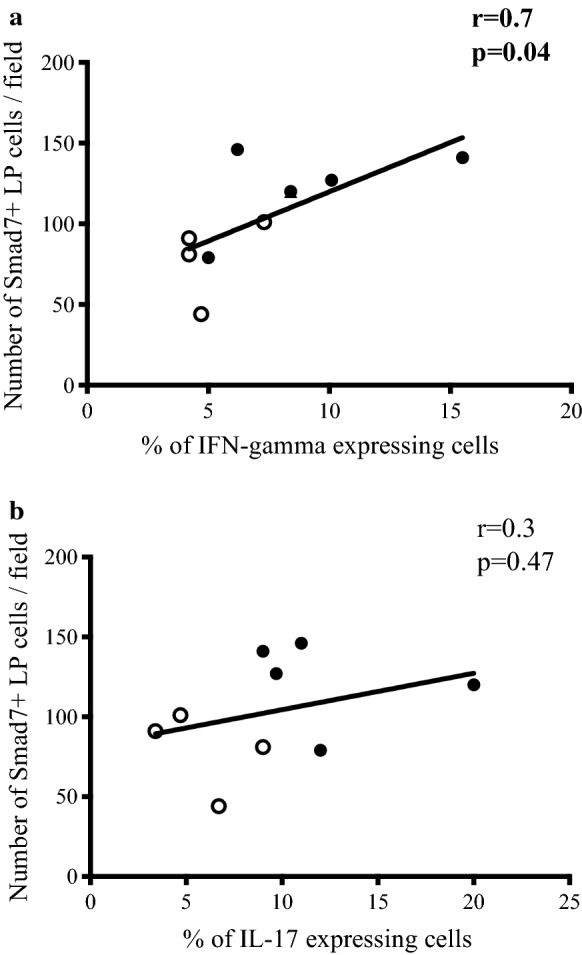
Smad7 expression correlates with the percentage of IFN-γ-producing cells in Crohn’s disease. Black circles and black dots respectively indicate CD patients with no endoscopic recurrence (i0–i1) and those with endoscopic recurrence (i2–i4). Correlation between the number of lamina propria Smad7-positive cells analysed by immunohistochemistry and the percentage of IFN-γ-positive cells **a** and IL-17A-positive cells **b** analysed by Flow cytometry in CD patients with no endoscopic recurrence (i0–i1) and CD patients with endoscopic recurrence (i2–i4). After curative ileo-colonic resection Smad7 positive cells correlates with the number of IFN-γ-secreting cells in the neo-terminal ileum of CD patients

We previously showed that the early phases of CD are marked by distinct mucosal profiles of cytokines. [8] In particular, before the appearance of endoscopic lesions, the mucosa of the neo-terminal ileum contains elevated levels of Th1-related cytokines and slightly increased IL-17A expression, while transition from this stage to endoscopic recurrence is characterised marked by abundance of Th1 cytokines and marked increase in IL-17A. Therefore, we assessed whether expression of Smad7 correlated with the number of cytokine-secreting cells. A significant correlation was found between the Smad7 expression and the number of IFN-γ-secreting cells but not with the number of IL-17A-producing cells (Fig. 4).

## Discussion

This study was undertaken to further assess the role of Smad7 in CD, and particularly to examine whether Smad7 is induced in the initial an/or late phases of the disease. Our previous study showed that few months after a curative ileo-colonic resection the mucosa of the neo-terminal ileum of CD patients is massively infiltrated with cytokine-secreting T cells and macrophages. [8] This occurs independently of the presence of endoscopic recurrence and therefore can be considered as “a very early stage” of the lesions. We collected biopsy samples from the neo-terminal ileum of CD patients 6 and/or 12 months after the surgery and examined Smad7 expression by immunohistochemistry. Smad7-expressing cells were abundant in the mucosa of CD patients as compared to controls and this was evident at each time point analysed. In particular, Smad7 was induced early after the intestinal resection and its expression was maintained at high level during the course of the disease. In line with our previous studies, [13, 22] Smad7 was expressed by both epithelial cells and lamina propria mononuclear cells, and in each of these compartments the levels of the protein were greater than those seen in the unaffected mucosa of control patients.

It is widely known that Smad7 exerts its regulatory effect on TGF-β activity in the cytoplasm, where the protein can interact and modulate the function of several molecules with the down-stream effect of inhibiting TGF-β/Smad signalling. [15] Immunohistochemical analysis of CD sections showed that Smad7 accumulated in both the cytoplasm and nucleus of epithelial cells and LPMC. This raises the possibility that Smad7 can have an additional and yet unidentified nuclear function. In this context, it has been recently shown that a short natural splice form of the deubiquitinating enzyme CYLD (sCYLD), a tumour suppressor that is mutated in patients with familial cylindromatosus, interacts with Smad7 in the nucleus of CD mucosal T cells, where the complex inhibits the binding of Smad3 to the DNA, thereby abrogating TGF-β activity. [23].

Enhanced expression of Smad7 in the initial, histological phases of CD could have important implications for the propagation of the inflammatory events, which lead to the development of the endoscopic recurrence. This hypothesis is supported by the demonstration that Smad7 expression in T cells correlates with disease severity in patients with CD [23] and mice overexpressing sCYLD and Smad7 develop spontaneous colitis due to altered TGF-β signalling and mediated by excessive activation of effector T cells.[23] These latter findings support and expand on data of our previous studies showing that inhibition of Smad7 in the gut of mice with experimental colitis restores TGF-β signalling thus suppressing cytokine responses and limiting the ongoing colitis [24].

Among the many cytokines produced within the inflamed tissue of CD patients, IFN-γ is supposed to trigger inflammatory pathways, which are relevant for CD pathogenesis. [25] Notably, production of this cytokine is negatively regulated by TGF-β [25] and therefore it is not surprising that restoring TGF-β signalling with Smad7 AS associated with down-regulation of IFN-γ production. Data of the present study further supports this notion as in the early stage of CD the number of Smad7-expressing cells was positively correlated with the number of IFN-γ-producing cells.

We are aware that the relatively small sample size can represent a limitation of this study, even though there was a noticeable difference between CD patients and controls in terms of Smad7, and the induction of Smad7 was consistently increased in CD independently of the phase of the lesions.

## Conclusions

Our study shows that Smad7 induction is an early event in the inflammatory sequence occurring in CD. This finding could have some potential therapeutic implications, as knockdown of Smad7 could help prevent post-operative recurrence in CD patients.

## Supplementary information

**Additional file 1:** **Table S1.** Current therapy, endoscopic lesions and number (N) of Smad7-positive cells in the lamina propria (LP) and in the epithelium compartment for each patient at each time-point.

## Data Availability

The datasets used and/or analysed during the current study are available from the corresponding author on reasonable request.

## Abbreviations

CD: Crohn’s Disease
IBD: Inflammatory bowel disease
Th: T-helper
TNF-α: Tumor necrosis factor alpha
TGF-β1: Transforming growth factor
AS: Antisense oligonucleotide
CTR: Control
DAB: Diaminobenzidine
HPF: High power field
LPMC: Lamina propria mononuclear cells
FBS: Fetal bovine serum
FACS: Fluorescent-activated cell sorter
PMA: Phorbol myristate acetate
IL: Interleukin
IFN: Interferon
CDAI: Crohn disease clinical index
sCYLD: Splice form of the deubiquitinating enzyme CYLD
N: Number
