# Supplementary material for: High Smad7 in the early post-operative recurrence of Crohn’s disease
Source: J Transl Med. 2020 Oct 19;18:395. doi: 10.1186/s12967-020-02558-8 (PMC7574182; doi:10.1186/s12967-020-02558-8)
Supplement: Supplementary file 1 — Additional file 1: Table S1. Current therapy, endoscopic lesions and number (N) of Smad7-positive cells in the lamina propria (LP) and in the epithelium compartment for each patient at each time-point. [file 12967_2020_2558_MOESM1_ESM.docx]

| **Pts** | **Surgery** | | | **Endoscopy at 6 months** | | | | **Endoscopy at 12 months** | | | |
| --- | --- | --- | --- | --- | --- | --- | --- | --- | --- | --- | --- |
|  | Therapy | N. of Smad7+ LP cells | N. of Smad7+ epithelial cells | Rutgeerts score | Therapy | N. of Smad7+ LP cells | N. of Smad7+ epithelial cells | Rutgeerts score | Therapy | N. of Smad7+ LP cells | N. of Smad7+ epithelial cells |
| CD 1 | steroids | 113 | 118 | i2 | mesalamine | 101 | 146 | i4 | mesalamine | 75 | 97 |
| CD 2 | anti TNF-α | 97 | 91 | i1 | mesalamine | 45 | 93 | i3 | mesalamine | 58 | 41 |
| CD 3 | steroids | 75 | 54 | -- | mesalamine | -- | - | i2 | mesalamine | 44 | 70 |
| CD 4 | steroids | 132 | 116 | i4 | mesalamine | 86 | 154 | i4 | azathioprine | 91 | 140 |
| CD 5 | -- | -- | -- | i2 | mesalamine | 144 | 63 | i4 | mesalamine | 206 | 103 |
| CD 6 | steroids+ azathioprine | 84 | 133 | i2 | mesalamine | 120 | 139 | -- | -- | -- | -- |
| CD 7 | anti TNF-α | 120 | 92 | i4 | mesalamine | 146 | 97 | -- | -- | -- | -- |
| CD 8 | steroids+ azathioprine | 159 | 62 | i2 | mesalamine | 139 | 149 | i2 | mesalamine | 79 | 173 |
| CD 9 | -- | -- | -- | i0 | mesalamine | 111 | 184 | i2 | mesalamine | 81 | 150 |
| CD 10 | steroids | 141 | 77 | i4 | mesalamine | 133 | 98 | i4 | azathioprine | 127 | 163 |
| CD 11 | -- | -- | -- | -- | -- | -- | -- | i2 | mesalamine | 176 | 134 |
| CD 12 | -- | -- | -- | -- | -- | -- | -- | i2 | mesalamine | 121 | 127 |
| CD 13 | steroids | 87 | 99 | i2 | mesalamine | 105 | 174 | -- | -- | -- | -- |
| CD 14 | steroids | 125 | 60 | -- | mesalamine | -- | -- | i4 | mesalamine | 141 | 99 |
| CD 15 | steroids | 63 | 99 | i0 | mesalamine | 110 | 158 | i0 | mesalamine | 81 | 169 |
| CD 16 | -- | -- | -- | i0 | no medication | 64 | 167 | -- | -- | -- | -- |
| CD 17 | -- | -- | -- | -- | mesalamine | -- | -- | i0 | mesalamine | 83 | 136 |

Supplementary Table1. Current therapy, endoscopic lesions and number (N.) of Smad7-positive cells in the lamina propria (LP) and in the epithelium compartment for each patient at each time-point.
